# A Modeling and Machine Learning Pipeline to Rationally Design Treatments to Restore Neuroendocrine Disorders in Heterogeneous Individuals

**DOI:** 10.3389/fgene.2021.656508

**Published:** 2021-09-09

**Authors:** Tongli Zhang

**Affiliations:** Department of Pharmacology and Systems Physiology, College of Medicine, University of Cincinnati, Cincinnati, OH, United States

**Keywords:** neuroendocrine dysfunction, stress, depression, machine learning, computational psychiatry, computational modeling, post-traumatic stress disorder

## Abstract

Heterogeneity among individual patients presents a fundamental challenge to effective treatment, since a treatment protocol working for a portion of the population often fails in others. We hypothesize that a computational pipeline integrating mathematical modeling and machine learning could be used to address this fundamental challenge and facilitate the optimization of individualized treatment protocols. We tested our hypothesis with the neuroendocrine systems controlled by the hypothalamic–pituitary–adrenal (HPA) axis. With a synergistic combination of mathematical modeling and machine learning (ML), this integrated computational pipeline could indeed efficiently reveal optimal treatment targets that significantly contribute to the effective treatment of heterogeneous individuals. What is more, the integrated pipeline also suggested quantitative information on how these key targets should be perturbed. Based on such ML revealed hints, mathematical modeling could be used to rationally design novel protocols and test their performances. We believe that this integrated computational pipeline, properly applied in combination with other computational, experimental and clinical research tools, can be used to design novel and improved treatment against a broad range of complex diseases.

## Introduction

Proper response to stress signal is essential to maintain the physiological and psychological health. Upon the stimulation by stress signals, the corticotropin-releasing hormone (CRH) is released from the hypothalamus and results in the release of adrenocorticotropic hormone (ACTH). Through the circulation system, ACTH then travels to the adrenal glands, binds to ACTH receptors, and stimulates the secretion of corticosteroids such as cortisol. Cortisol then stimulates the increases of glucose concentration in the blood to provide energy to cope with the stresses. The proper functioning of this hypothalamic–pituitary–adrenal (HPA) axis is important for physiological response to stress (Tsigos and Chrousos, 2002; Dunlop and Wong, 2019); while the dysregulation of the HPA axis is closely associated with stress order, such as post trauma stress disorder (PTSD) and depression (Bisson et al., 2015; Yehuda et al., 2015; Shalev et al., 2017). If the dysregulated dynamics of the HPA axis is reversed and the normal dynamics and function of the HPA axis were restored, it might help with treating stress disorders (Ronaldson et al., 2018; Menke, 2019).

However, the effective restoration of HPA function is challenged by the heterogenous dynamics of the dysregulated axis in patients with stress disorders. For example, in patients with PTSD, both lower cortisol levels and higher cortisol have been reported. In patients with other stress disorders, the cortisol levels are also reported to be bimodal (Yehuda et al., 1995; Gold and Chrousos, 2002; Bremner et al., 2007; Meewisse et al., 2007).

To cope with this challenge of heterogeneity and facilitate the optimization of treatment protocols that can effectively restore HPA axis dynamics, we explored the potential of an integrated computational pipeline that combines mathematical modeling and machine learning. The computational model incorporates several feedbacks controlling the HPA axis, with which we can computationally scanned the effects of potential targets. Machine learning analysis of the random scanning results then revealed the effective targets and how these targets should be perturbed. These ML derived insights aided us to design novel, optimized treatment protocols, which could be further tested with mathematical models.

Our analysis demonstrated a “*proof of concept*” that an integration of mathematical modeling and machine learning can be used to efficiently explore a heterogeneous patient population and facilitate the design of optimized treatment protocols. In the discussion, we have also commented on the strength and limitation of this computational pipeline and envisioned how it could be used together with other tools to improve clinical treatments of complex diseases.

## Materials and Methods

### Time Series Simulation

Simulations were carried out using the ordinary differential equations built following the standard formula. All parameter values were randomly selected from uniform distributions of broad ranges. Time series simulations were performed using XPPaut,^1^ the simulated data were then plotted using Matlab.^2^ The detailed simulation protocols for each figure was described along with the figure.

### Classification Tree Analysis

Tree models were run using the model parameters in addition to the steady state values for model components. Trees were computed in R^3^ using the rpart2 algorithm.

### Random Forest Analysis

Random Forest analysis was carried out with the value change of parameters as input features and the outcome (effective or non-effective) as prediction targets. The analysis was performed using the standard package in R (see footnote 3). Permutation feature importance were scaled to the maximum (100%) and plotted.

### Implementation of Treatments

All treatments were implemented as transient changes of the model parameters. In the random against random targets, a random target parameter is chosen and then either increased or decreased by a value between zero and 10. In the targeted treatments, the parameters with the top rank were decreased.

### Selection of Parameter Ranges

If the ranges of parameter changes were too small (i.e., 5 or 10%), the small changes of parameters result in mostly homogeneous behaviors and the sampling of heterogeneous response was computationally inefficient. With trails and errors in preliminary exploration, we chose all parameters randomly from uniform distributions that ranged 10-fold up and down their basal values (10–1,000%) to sample heterogenous responses efficiently. Since the patient behavior of interest were already covered by the current ranges, the ranges of the parameter changes were not further expanded.

## Results

### A Mathematical Model Integrated One Negative Feedback and Two Positive Feedbacks Controlling the HPA Axis

The HPA axis is characterized by a negative feedback: after the increase of the stress signal results in the sequential release of corticotropin-releasing hormone (CRH), adrenocorticotropic hormone (ACTH), and cortisol, the activated glucocorticoid receptors then represses both CRH and ACTH. This negative feedback has been implemented in previous mathematical models (Gudmand-Hoeyer et al., 2014; Bangsgaard and Ottesen, 2017; Stanojević et al., 2018a).

In addition, the glucocorticoid receptor is characterized by a positive feedback that potentially results in bistable switching (Sriram et al., 2012). Meanwhile, Kim et al. (2016) proposed the positive feedback regulating corticotropin-releasing hormone (CRH) could also result in switch like behavior.

Our current mathematical model has integrated the above mentioned negative and positive feedbacks to generate complex and heterogeneous dynamics, which serve as ideal tests to examine whether our analysis pipeline works. Since both the machine learning tools and modeling tools in our pipeline are applicable to systems with additional components, we expect that our analysis pipeline will be able to continue to provide useful and realistic insights even if the model of the HPA axis is expanded to incorporates more regulatory details than these three feedbacks.

### Facilitating the Exploration of Heterogeneity With a Standard Model Formula

The heterogeneity of different individuals could be implemented with different model structures, different model parameter values, or both. However, it would be computational expensive to explore the heterogeneity by composing a different set of mathematical equations for every single individual. In order to facilitate the exploration of heterogeneity and reduce the computational expense of the computational pipeline, we have adopted a standard formula to describe the model structure. In this way, we can simply change the parameter values to explore the heterogeneity between individuals.

In this standard formula, the dynamics of each model component (x) is described as:$\frac{dX_{i}}{dt}=\tau_{i}\;\left(F_{i}-X_{i}\right)$, with $F_{i}=\frac{1}{1+e^{-\sigma \;W_{i}}\;}\;$ and $W_{i}=R_{i}^{0}+\underset{j}{\sum }R_{i}^{j}\cdot X_{j}\;\;$.

In which, *τ_i_* describes the time scale of the component change, *F_i_* describes the steady state level of the component, and *W_i_* descries the net regulation received by the component.

In this formula, a positive $R_{i}^{j}$ specifies an activating effect, while a negative $R_{i}^{j}$ specifies an inhibitory one. $R_{i}^{0}$ sums effects that origin from all other components not explicitly incorporated in the model, and this parameter can be replaced with additional regulatory terms when the model is expanded. Additional elaboration of this approach and its dynamical properties are available in the literature (Mjolsness et al., 1991; Tyson and Novak, 2010) and our previous publication (Ballweg et al., 2018).

**Figure 1:**
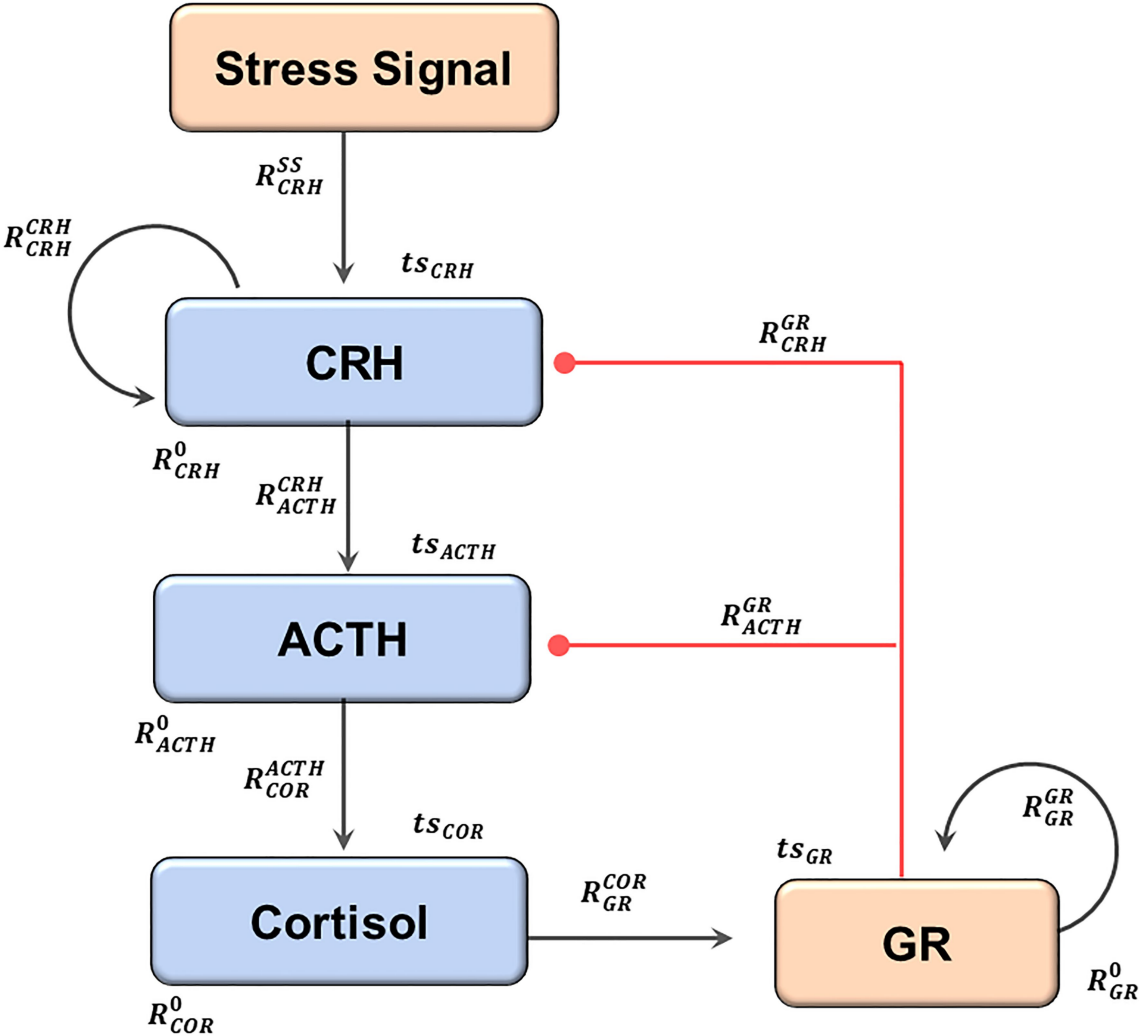
The structure of the current model. An elevation of the stress signal results in the sequential release of CRH, ACTH, and Cortisol. Cortisol activates GR, which then inhibits both CRH and ACTH, forming the negative feedback. Two positive feedback loops regulate CRH and GR. The black arrows indicate activation, and the red lines with dot heads indicate inhibition. The regulatory parameters (capital R) and the time series parameters (lower case ts) are labeled near the reactions they control. The subscripts describe the identities of the regulated components, and the superscripts describe the regulating components (0 if not specified). The full names of the components, as well as the modeling justifications, are elaborated in the text.

By changing the values of the regulatory parameters (Rs), we could conveniently explore the nature (activation or inhibition) as well as the strength of the interactions between the model components. The model simulations then allowed us to explore the dynamical properties of the HPA axis that resulted from the different interactions. The parameters explored in this work and the interaction they regulated have been illustrated in Figure 1, the regulatory roles of the parameters are described in Table 1. The ordinary differential equations and the initial values of the model parameters are recorded in the Supplementary Table 1.

**Table 1:** The matrix of model parameters.

Regulator	8 Regulatory parameters			
Stress signal	$R_{CRH}^{SS}$			
CRH	$R_{CRH}^{CRH}$	$R_{ACTH}^{CRH}$		
ACTH			$R_{COR}^{ACTH}$	
Cortisol				$R_{GR}^{COR}$
GR	$R_{CRH}^{GR}$	$R_{ACTH}^{GR}$		$R_{GR}^{GR}$
Component	CRH	ACTH	Cortisol	GR
8 Component specific parameters				
Background	$R_{CRH}^{0}$	$R_{ACTH}^{0}$	$R_{COR}^{0}$	$R_{GR}^{0}$
Time scale	*ts_CRH_*	*ts_ACTH_*	*ts_COR_*	*ts_GR_*

### Fractional Development of Stress Disorders Within Heterogeneous Individuals

With the standard model formula, we then mimicked a population of heterogeneous individuals by assigning random values to the control parameters. The dynamical simulations of the models represented the dynamical responses of these individuals to stress signals.

**Figure 2:**
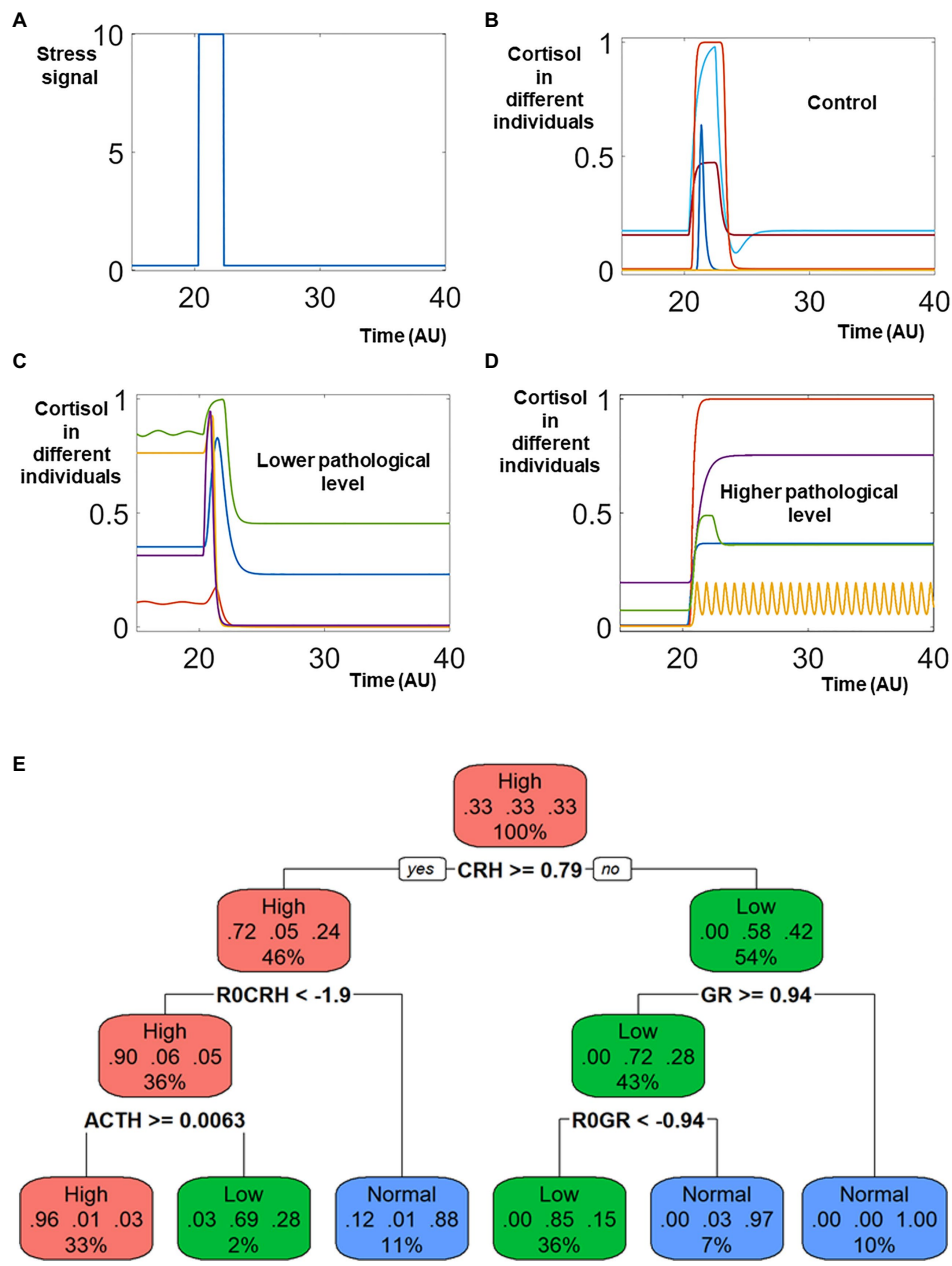
Sampling the heterogenous response triggered by stress signal. Identical model structure and different values of all parameters are used to simulate the heterogenous response to stress signal. In response to a transient elevation of the stress signal (around time 20, **A**), the cortisol levels in the control population **(B)** returned to normal levels after transient elevation, representing the healthy population who do not develop neuron endocrine disorder after stress. On the contrary, the levels of cortisol decreased and were sustained lower in patient populations who were characterized by lower pathological levels of cortisol **(C)**. The cortisol levels were elevated and remained higher in patients whose pathological cortisol levels were higher after stress **(D)**. **(E)** Classification Tree. The different colors of different nodes indicate the types of dominant populations: red nodes indicates that most individual in the node had higher cortisol after stress; green nodes indicates that most individuals were characterized by lower levels of cortisol; blue nodes indicate the ones with most individuals from the control population. In the top node, individuals from these three populations were of identical number, and the node is labeled red.

A transient elevation of the stress signal (Figure 2A) was applied to every individual within the heterogeneous population. In response to this increase of the stress signal, the cortisol levels were transiently elevated in almost all individuals. For the control population, the cortisol levels returned to base line after the stress signal decreased (Figure 2B), representing the return to the physiological homeostasis. On the other hand, the cortisol levels sustained at either lower or higher levels represented patients with stress disorders (Figures 2C,D).

An analysis with the classification and regression tree (CART) provided an overview of features (including both model component and parameters) characterizing these three different populations (Figure 2E). The high cortisol patients are characterized by high levels of CRH and ACTH, which makes mechanistic sense since CRH and ACTH promote cortisol release in the HPA axis. The lower cortisol patients, on the other hand, are characterized by low ACTH and high level of GR. The high level of GR in these patients repress their cortisol release.

The simulated dynamics indicates that the systems changes might be sustained even after the decrease of stress signal, which is a hallmark of post trauma stress disorder (PTSD). Upon the exposure to transient traumatic events, the symptoms in PTSD patients could sustain for a long time. Many military personnel suffer from stress disorder years after departing from the battle field (O'Toole et al., 2009; Marmar et al., 2015; Armenta et al., 2018).

Our simulations used a strong stress signal, which was necessary to trigger cortisol disorders. If the stress signal were reduced, less simulated individuals developed cortisol disorder. Meanwhile, even for the strong stress signal used in our simulation, no all the affected individuals developed cortisol disorder. Rather, a large portion of the stimulated individuals (>50%) could recover their physiological cortisol levels after the stress disappears. The low percentage of stress disorder development was also reported in the literature. The World Health Organization World Mental Health Surveys reported a cross-national lifetime prevalence rate of 3.9% (Koenen et al., 2017); while a National Epidemiologic Survey reported a lifetime prevalence rate of 6.1% in the United States (Goldstein et al., 2016).

Our simulations indicated that the heterogeneous levels of cortisol, either lower or higher, might naturally emerge after heterogenous individuals were stimulated with stress signal. The heterogenous cortisol levels in the patient populations were consistent to the literature reports (Yehuda et al., 1995; Gold and Chrousos, 2002; Bremner et al., 2007; Meewisse et al., 2007). The heterogeneous levels of cortisol made sense when we examined the structure of the mathematical model. The level of cortisol was regulated by a combination of two positive feedbacks and one negative feedbacks. The positive feedbacks allowed the model to have the potential to generate different attracting stable steady states, which potentially could explain the distinction between the higher cortisol population, the lower cortisol population, and the control.

Meanwhile, the negative feedback had the potential to generate sustained oscillations (one example illustrated in Figure 2D). The regulation and dysregulation of the oscillatory dynamics of cortisol might also play a role in the response to stress stimulation (Kalafatakis et al., 2018; Stanojević et al., 2018b; Lightman et al., 2020).

With the intervened positive and negative feedbacks, the current model has been able to mimic the complex, heterogeneous dynamics of different individuals who develop stress disorder, hence we proceed to use the current model to test our integrated computational pipeline.

### Random Scanning of Treatment Targets

**Figure 3:**
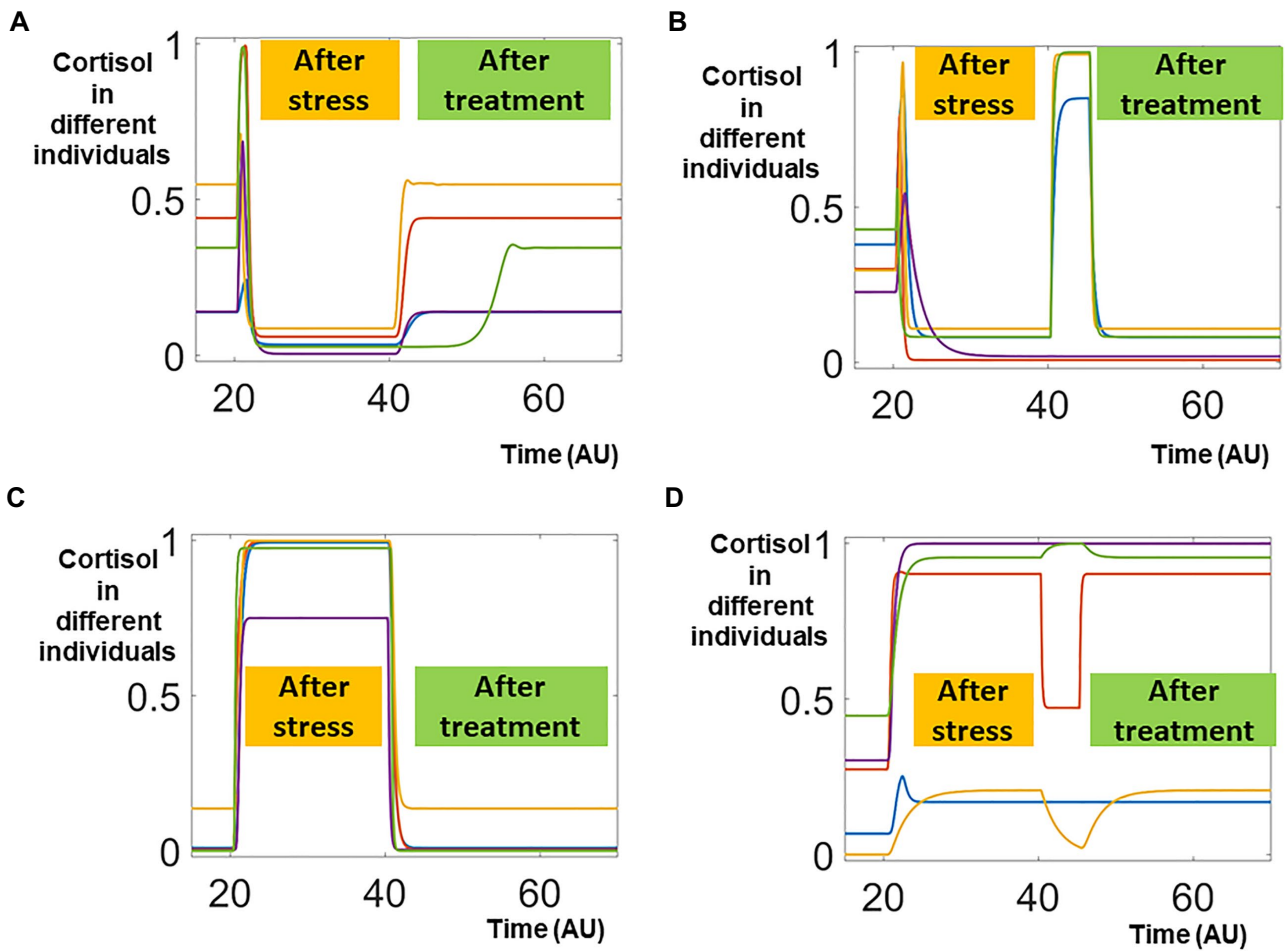
Sampling effective and non-effective treatments. In this random scanning, one parameter is randomly perturbed around time 40. The effective treatments, which restored the cortisol distorted by the stress signal, were shown in panels **(A,C)**. On the other hand, the non-effective treatments, which failed to restore the cortisol levels, were shown in panels **(B,D)**.

We first selected around 30,000 patients with lower pathological cortisol and subjected them to for potential treatments that were applied on the control parameters. Since we lacked both qualitative information (what targets should be targeted) as well as quantitative information (to what levels should the targets be changed), we randomly selected an individual parameter target and changed its level randomly and transiently (details in Materials and Methods). Five representative trajectories of effective treatments were illustrated in Figure 3A. These five individuals started with normal, physiological levels of cortisol. After transient stimulation by the stress signal (applied around time 20), their cortisol levels were decreased to lower, pathological levels. After the effective treatments, their cortisol levels were restored to normal, physiological ones. On the contrary, the cortisol levels were not restored in five individuals representing patients receiving ineffective treatments (Figure 3B).

Such bimodal responses were also observed in around 30,000 individuals whose pathological cortisol levels were higher than their physiological ones. In five representing individuals whose cortisol levels were sustained higher after the transient stress signal, effective treatment restored their cortisol levels to physiological ones (Figure 3C). On the other hand, the cortisol levels remained higher after ineffective treatments (Figure 3D).

Treatments against random targets were effective in small portions of the patients but ineffective in the majorities of the treated individuals. For our pipeline, even the ineffective treatments provided important information on how the system responded to perturbations. Hence, proceeded to apply machine learning analysis on the simulation data that included both effective and ineffective treatments. In the future, we envision that our pipeline could potentially be applied to the clinical data that combined effective and ineffective treatments, to improve the design of clinical trials and treatment protocols.

### Improving the Treatment Protocols

The individual responses to random treatments were binary (Effective vs. Non-Effective), and such binary data were fed into the Random Forest (RF) analysis. RF identified the most influential factors that distinguished the effective treatments from the non-effective ones. By computing the consequential error resulted from permuting features, the RF analysis also ranked the relative importance of all potential the targets.

**Figure 4:**
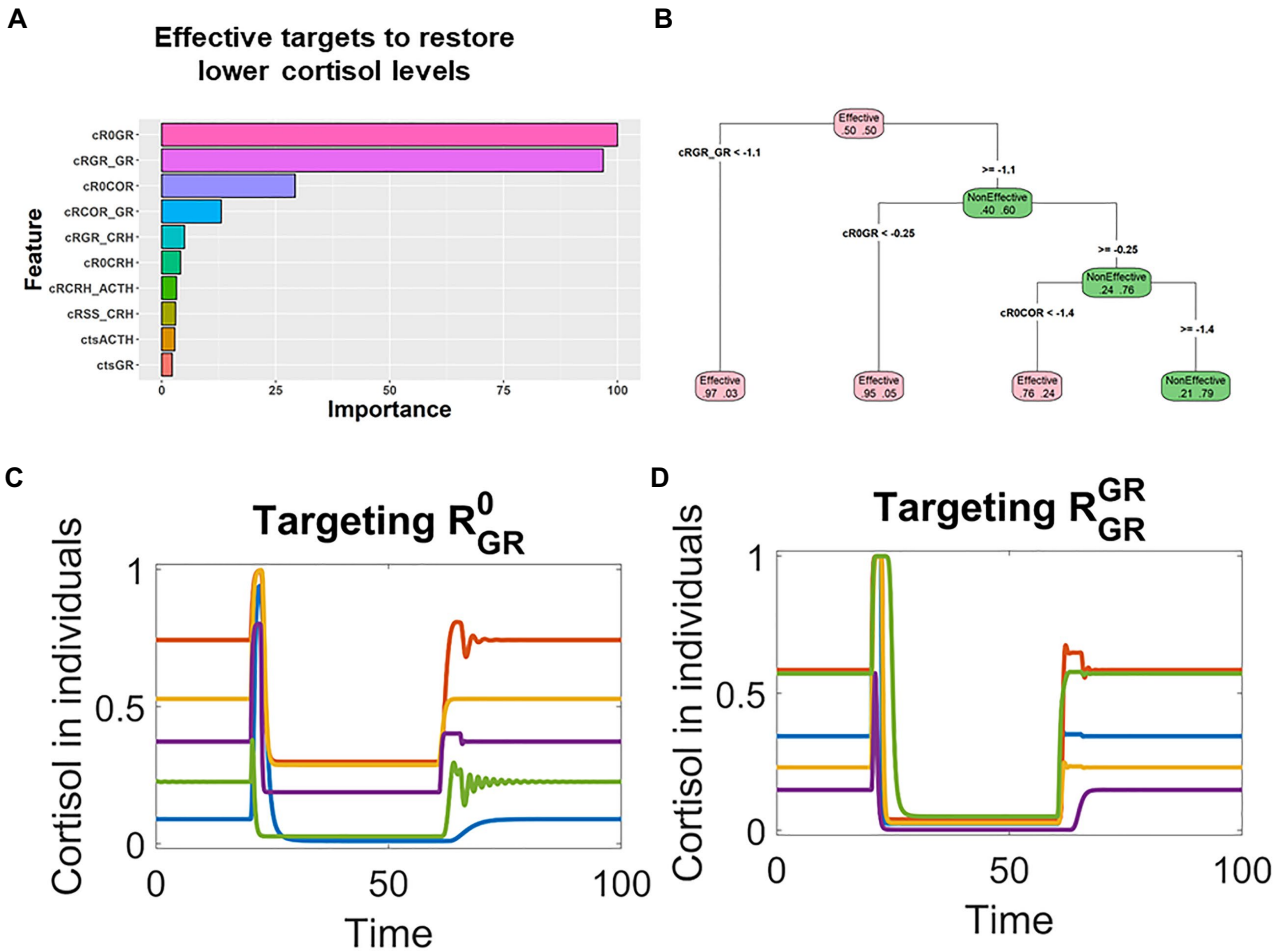
Machine learning analysis revealed that the key parameters controlling the GR positive feedback were associated with effective treatments for patients with lower cortisol levels. **(A)**. Random forest analysis ranked the targets based on their association with the effective treatments in patients whose pathological cortisol levels were lower. **(B)**. Decision tree analysis indicated that these parameters were decreased in effective treatments. **(C,D)** Time series simulations illustrated how the decrease of $R_{GR}^{0}$ or $R_{GR}^{GR}$ restored the cortisol levels in patients with lower pathological cortisol levels.

The RF analysis revealed that for individuals whose pathological cortisol levels were lower, the changes of $R_{GR}^{0}$ and $R_{GR}^{GR}$ were most important for effective treatments (Figure 4A). Furthermore, the analysis with decision tree provided quantitative information on how the targets should be changed (Figure 4B). In the treatments that sufficiently decreased the level of $R_{GR}^{GR}$, most of them would be effective (node on the bottom left in Figure 4B); similarly, most of the treatments that decreased the level of $R_{GR}^{0}$ were also effective (2nd node from left on the bottom of Figure 4B).

These RF identified targets, $R_{GR}^{0}$ and $R_{GR}^{GR}$, make mechanistic sense in the context of the model structure. A positive feedback controlling GR might result in pathological steady state with lower cortisol levels. Hence, it is reasonable to expect that the pathological states could be reverted by targeting the key parameters that control the positive feedback. On the basis of these qualitative and quantitative information, we designed targets treatments against the top targets, $R_{GR}^{0}$ or $R_{GR}^{GR}$. Simulation showed that transient decrease of these two targets were able to effectively treat patients whose pathological cortisol levels were lower. These patients were characterized with lower levels of cortisol after their stimulation by transient stress signal (applied around time 20). Then, their cortisol levels were restored by the transient decrease of $R_{GR}^{0}$ or $R_{GR}^{GR}$ applied around time 60 (Figures 4C,D).

**Figure 5:**
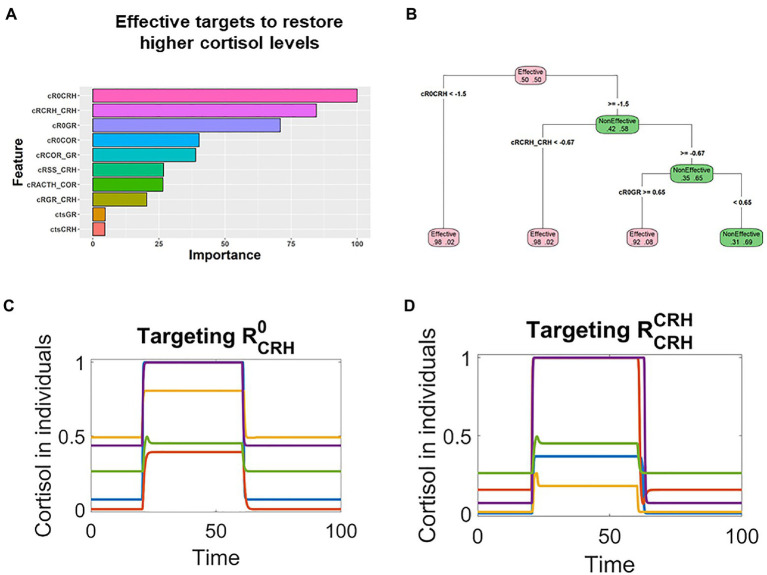
Machine learning analysis revealed that parameters controlling the CRH positive feedback were associated with effective treatments for patients with higher cortisol levels. **(A)** Random forest analysis ranked the targets based on their association with the effective treatments in patients whose pathological cortisol levels were higher. **(B)** Decision tree analysis indicated that these key parameters should be decreased for the treatments to be effective. **(C,D)** Time series simulations illustrated how the decrease of $R_{CRH}^{0}$ or $R_{CRH}^{CRH}$ restored the cortisol levels in patients who had higher levels of cortisol after stress stimulation.

Similarly, RF analysis indicated that $R_{CRH}^{0}$ and $R_{CRH}^{CRH}$ were the top targets in patients whose pathological cortisol levels were higher (Figure 5A). The decision tree analysis suggested that these two targets should be decreased in effective treatments (Figure 5B), and rationally designed treatments based on such information could effectively restore the levels in these patients. After stimulation by transient stress signal (applied around time 20), cortisol levels were sustained higher in these individual patients. Then, $R_{CRH}^{0}$ and $R_{CRH}^{CRH}$ were transiently decreased around time 60, the cortisol levels were decreased and remained low (Figures 5C,D).

It was encouraging that the treatments effects were sustained even though the treatments against these highly ranked targets were only transient. In clinical terms, this means that it would be possible to fully cure the patients suffering stress disorder if the correct targets were identified and perturbed.

Hence, starting with scanning treatments against random targets, machine learning analysis with the scanning results could lead us to rationally design novel and improved treatments. What is more, since the machine learning analysis revealed more than one target that could contribute to the effective treatments, it would be plausible to design multiple effective treatments and select the practical ones based on clinical constrains.

## Discussion

Heterogeneity within individual patients underlies partial responses to treatment and calls for the design of personalized and optimized treatment protocols. In this work, we have demonstrated the performance of a computational pipeline that integrated mathematical modeling and machine learning. The pipeline was able to address this fundamental challenge of heterogeneity: starting with little qualitative clue (target identification) and quantitative clue (perturbation strength), the pipeline was able to deliver rational designs of effective treatment plans that clearly answered ***“what to targets?” and “how much to change?”***

With such “*proof of principle*,” we hope that the computational pipeline could be integrated into clinical practice to design novel and more effective treatments for complex diseases. We envision that after clinical data were fed into this analysis pipeline, it would lead to insights that are clinically applicable.

**Figure 6:**
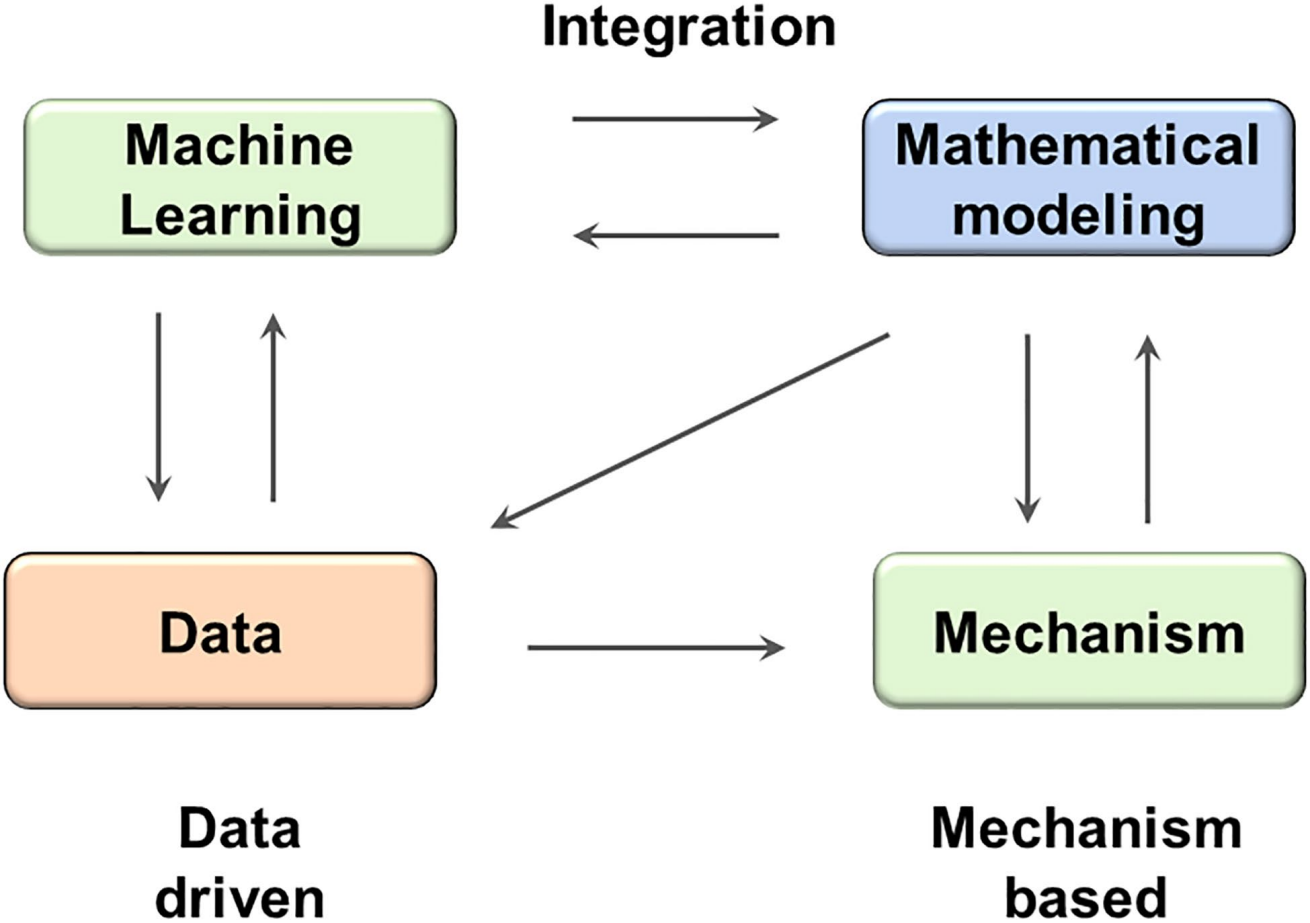
The integration between machine learning and mathematical modeling might result in synergistic effect. ML methods can facilitate the analysis of both real data and simulated data generated by mathematical models; while mathematical models can test hypothesis provided by ML models and reveal mechanistic insights.

Two theoretical approaches, one driven by data and the other based on mechanism, have been widely applied in the field of systems biology and quantitative systems pharmacology. In this work, we have illustrated that these two approaches could be integrated together to achieve synergistic effects (Figure 6): the machine learning methods could be used to efficiently extract insights from heterogeneous behaviors, while the mechanistical models could be used to design mechanistic and dynamical protocols that are directly translatable on the basis of the machine learning revealed insight.

The machine learning methods could help modeling to be more efficient. It would be computationally expensive to carry out a comprehensive scanning with all possible targets and all possible perturbation values, in comparison, a random scanning followed by machine learning analysis in our work was able to efficiently yield hints on some top ranked targets and how they should be perturbed. Meanwhile, modeling puts the insights extracted with machine learning back into the mechanistic context and could help designing novel protocols directly translatable to clinical practice.

Consistent with its purpose of showing a “*proof of principle*,” the current model has represented the heterogeneous patient populations only with the qualitative changes (either lower or higher) of their cortisol levels. As the next step, it would be beneficial to fit the model parameters with cortisol levels observed in individual patients (Bangsgaard and Ottesen, 2017), so that the heterogeneous models could represent individual patients in quantitative details.

With the continuous contribution from the communities of systems biology and quantitative systems pharmacology, we expect the simplified model would be expanded to more realistic, multi-scale ones that include more biochemical, genetic, epigenetic, molecular, cellular, and neurological details. This process would be time and effort consuming; however, the overall process could be facilitated by taking advantage of the existing models that have been developed to describe HPA axis and its role in stress orders. For example, our current work has been benefiting from the modeling works or Sriram et al. (2012) and Kim et al. (2016). We envision the further expansion of the current model will also be able to utilize many other modeling works in the field of computational psychiatry, such as the PKA-PP2A model of fear conditioning (Yang et al., 2010), the model for protein kinase M feedback (Ogasawara and Kawato, 2010), and the modeling work on the positive feedback loop controlling brain-derived neurotrophic factor (BDNF; Bambah-Mukku et al., 2014; Zhang et al., 2016). The incorporate of additional regulatory pathways might result in the co-existence of even more attractors, which would further increase the heterogeneous subtypes of stress disorder patients. Combination of different machine learning algorithms, including those used here, promises to facilitate the analysis of these additional subtypes.

In addition, we expect that the expanded models of stress disorders will also integrate multi-scale neural circuits within the corresponding regions of the brain (Smith, 2005), the pharmacokinetics of various drugs such as the selective serotonin reuptake inhibitors sertraline (Zoloft) and paroxetine (Paxil; Alhadab and Brundage, 2020; Heydorn, 1999), as well as the pharmacodynamic effect of these drugs such as the serotonin production and regulation (Best et al., 2010).

Though it is going to be time consuming and effort consuming to develop such realistic models with elaborated biological and pharmaceutical control details, we expect that the effort will eventually pay off and the realistic models will make contributions of clinical significance. For example, the realistic models may be able to guide us to further understand the genetic and biochemical basis of different patients whose cortisol levels are either lower or higher when developing depression; these models may point out to optimized targets for patients who are not responding to the currently available treatments; also, comprehensive models will have the potential to aid us to examine whether novel treatments would result in undesired side effects or toxicities in healthy, control populations.

Computational psychiatry promises to address some of the hard challenges faced by psychobiological researchers, and encouraging results have been accumulating along this direction (Ferrante and Gordon, 2021; Huys et al., 2021). From a methodological perspective, we have tested an integrated computational pipeline (ICP) that combines computational modeling and machine learning and shown “*proof of principle*” that this pipeline could be used to aid with the design of novel treatment protocols which can effectively restore neuroendocrine dysregulation in a population of heterogeneous individuals. We expect that the further expansion of the model as well as this pipeline would be able to deliver more clinically useful insights for psychological disorders.

What is more, this computational framework of integrated modeling and machine learning can be readily applied to other research areas beyond neuroendocrine and psychological disorders. The field of computational medicine and quantitative systems pharmacology have already started to integrate complimentary tools to achieve greater benefits (Hutchinson et al., 2019; Zhang et al., 2019; Benzekry, 2020), and we believe that the broader application of our pipeline will contribute to the design of novel and effective treatments for a board range of complex diseases.

## Data Availability Statement

The original contributions presented in the study are included in the article/Supplementary Material, further inquiries can be directed to the corresponding author.

## Author Contributions

The author confirms being the sole contributor of this work and has approved it for publication.

## Funding

The work was supported by grant 1016183 ARMY W911NF-20-1-0192 to TZ.

## Conflict of Interest

The author declares that the research was conducted in the absence of any commercial or financial relationships that could be construed as a potential conflict of interest.

## Publisher’s Note

All claims expressed in this article are solely those of the authors and do not necessarily represent those of their affiliated organizations, or those of the publisher, the editors and the reviewers. Any product that may be evaluated in this article, or claim that may be made by its manufacturer, is not guaranteed or endorsed by the publisher.

## Footnotes

^1^ http://www.math.pitt.edu/~bard/xpp/xpp.html

^2^ https://www.mathworks.com

^3^ https://www.r-project.org/

## Supplementary Material

Supplementary Table 1 The ordinary differential equations and basal values of the model parameters.
