# Supplementary material for: A Modeling and Machine Learning Pipeline to Rationally Design Treatments to Restore Neuroendocrine Disorders in Heterogeneous Individuals
Source: Front Genet. 2021 Sep 9;12:656508. doi: 10.3389/fgene.2021.656508 (PMC8458900; doi:10.3389/fgene.2021.656508)
Supplement: Supplementary Table 1 — The ordinary differential equations and basal values of the model parameters. [file Table_1.docx]

**Supplementary Table 1. The ordinary differential equations and basal values of the model parameters**

$$wCRH = R0CRH + RCRH\_CRH*CRH + RSS\_CRH*StreSig + RGR\_CRH*GR$$

$$FCRH = 1/(1+exp(-sigma*wCRH))$$

$$CRH' = tsCRH*(FCRH-CRH)$$

$$wACTH= R0ACTH+ RCRH\_ACTH*CRH + RGR\_ACTH*GR$$

$$FACTH= 1/(1+exp(-sigma*wACTH))$$

$$ACTH' = tsACTH*(FACTH-ACTH)$$

$$wCOR = R0COR + RACTH\_COR*ACTH$$

$$FCOR = 1/(1+exp(-sigma*wCOR))$$

$$COR' = tsCOR*(FCOR-COR)$$

$$wGR = R0GR + RCOR\_GR*COR + RGR\_GR*GR$$

$$FGR = 1/(1+exp(-sigma2*wGR))$$

$$GR' = tsGR*(FGR-GR)$$

Parameters (basal values)

par sigma=2, sigma2=5

par R0CRH=-0.5, R0ACTH=-0.5, R0COR=-0.5, R0GR=-0.5

par RSS_CRH=1, RCRH_ACTH=1, RACTH_COR=1, RCOR_GR=1

par RCRH_CRH=1, RGR_GR=1, RGR_CRH=-1, RGR_ACTH=-1

par tsCRH=1, tsACTH=1, tsCOR=1, tsGR=1
